# On estimation and identifiability issues of sex-linked inheritance with a case study of pigmentation in Swiss barn owl (*Tyto alba*)

**DOI:** 10.1002/ece3.1032

**Published:** 2014-03-29

**Authors:** Camilla T Larsen, Anna M Holand, Henrik Jensen, Ingelin Steinsland, Alexandre Roulin

**Affiliations:** 1 Department of Mathematical Sciences, NTNUNO-7491, Trondheim, Norway; 2 Department of Mathematical Sciences, Centre for Biodiversity Dynamics, NTNUNO-7491, Trondheim, Norway; 3 Department of Biology, Centre for Biodiversity Dynamics, NTNUNO-7491, Trondheim, Norway; 4 Department of Ecology and Evolution, University of Lausanne1015, Lausanne, Switzerland

**Keywords:** Approximate Bayesian animal model, barn owl (*Tyto alba*), integrated nested Laplace approximation, quantitative genetics, sex chromosome, sex-linked additive genetic effects

## Abstract

Genetic evaluation using animal models or pedigree-based models generally assume only autosomal inheritance. Bayesian animal models provide a flexible framework for genetic evaluation, and we show how the model readily can accommodate situations where the trait of interest is influenced by both autosomal and sex-linked inheritance. This allows for simultaneous calculation of autosomal and sex-chromosomal additive genetic effects. Inferences were performed using integrated nested Laplace approximations (INLA), a nonsampling-based Bayesian inference methodology. We provide a detailed description of how to calculate the inverse of the X- or Z-chromosomal additive genetic relationship matrix, needed for inference. The case study of eumelanic spot diameter in a Swiss barn owl (*Tyto alba*) population shows that this trait is substantially influenced by variation in genes on the Z-chromosome (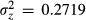 and 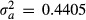). Further, a simulation study for this study system shows that the animal model accounting for both autosomal and sex-chromosome-linked inheritance is identifiable, that is, the two effects can be distinguished, and provides accurate inference on the variance components.

## Introduction

In general, quantitative genetic methods implicitly assume only autosomal inheritance when estimating variance components and heritability for different types of traits (Qvarnström *et al*. 2006; Foerster *et al*. 2007; Forstmeier *et al*. 2011). In this study, we explore the consequences of not modeling sex-linked inheritance when estimating additive genetic effects when some of the genes controlling a trait are located on a sex chromosome.

The heterogametic parent, for example, XY males in mammals, and ZW females in birds, only gives its X/Z sex chromosome to its homogametic offspring (i.e., XX females in mammals and ZZ males in birds). Hence, when the selection acts strongest on the heterogametic sex in the population, the genes on the X/Z sex chromosome will be exposed to selection only half of the time compared with genes on autosomes (Rice 1984). Thus, selection will influence genes located on the autosomes the most (Charlesworth *et al*. 1987), and as a result, we would expect to see a much slower change over time in genes on the sex chromosome than in genes located on the autosomes. How natural selection and sexual selection affect the evolution of a trait will depend on whether the contributing genes are on autosomes or sex chromosomes. The importance of determining whether a given gene, quantitative trait locus (QTL), or part of genetic variation that contributes to phenotypic variation is located on autosomes or sex chromosomes has been emphasized in many studies (Charlesworth *et al*. 1987; Mank and Ellegren 2009; Blackburn *et al*. 2010). This knowledge is especially important in understanding the evolution of sexual dimorphism (Rice 1984), but may also affect the rate and direction of phenotypic evolution in general (Lande 1980; Kirkpatrick and Hall 2004).

A generalized linear mixed model that offers a powerful approach to estimate genetic variance components, such as autosomal and sex-linked additive genetic variance, is the so-called animal model (Kruuk 2004). In contrast to simpler methods such as parent–offspring regression or sib designs, animal models utilize information from different relationships between individuals in large and complex pedigrees simultaneously. Animal models express the phenotypic value of a given trait as a linear sum of fixed and random effects, where the different random effects have a specified covariance structure. The most important structured random effect is the additive genetic effect (breeding value), which has a covariance structure given by the additive relationship matrix (Lynch and Walsh 1998). Including the additive genetic effect allows for estimation of important genetic parameters such as additive genetic variance and heritability.

However, the covariance structure of the breeding values reflects a mode where the genetic relationship between relatives of the same degree is assumed equal irrespective of sex, and as such it corresponds to an autosomal mode of inheritance (in that each individual inherits one half of its autosomal genes from each of its parents). This representation of the additive genetic effect does not take into account that sex-chromosomal genes might contribute substantially to the total additive genetic effect and variance for certain traits. For example, sex-linked effects are found in *Drosophila* (Cowley *et al*. 1986), in birds (Sætre *et al*. 2003), and humans (Pan *et al*. 2007).

The assumption of only autosomal inheritance may not only prevent one from gaining important knowledge about where the genes contributing to phenotypic variation are located, but may also result in inflated estimates of what should be interpreted as autosomal additive genetic variance. The latter occurs due to the similarities in the inheritance patterns (Grossman and Eisen 1989; Lynch and Walsh 1998), and thus the covariance structure of autosomal and sex-linked genes. Erroneously assuming that all additive genetic variance is due to genes on autosomes may result in biased predictions for the rate and direction of adaptive evolution (Lande 1980; Kirkpatrick and Hall 2004).

To separate autosomal from sex-linked additive genetic variances using the animal model, we need to explicitly model sex-linked effects by utilizing the corresponding covariance structure of genes on the sex chromosomes. The theory on how to construct the necessary covariance matrix for inclusion of Z-linked additive effects is presented in Fernando and Grossman (1990).

However, only a few authors within evolutionary quantitative genetics have considered sex-linked additive genetic effects within the animal model framework. Fairbairn and Roff (2006) suggested to use the animal model for estimating genetic variance due to sex-linked genes in the context of evaluating of sexually dimorphic traits, yet they did not present any results from the proposed model. An extensive version of the animal model was presented in Meyer (2008), which, among other genetic and environmental effects, also accounted for sex-linked additive effects. They used simulated data on an experimental design to estimate the variance components using restricted maximum likelihood (REML) methods, and their results showed that the model was able to disentangle additive genetic variances caused by sex-linked and autosomal effects. To the best of our knowledge, Roulin *et al*. (2010) and Husby *et al*. (2013) are the only authors who have applied an animal model accounting for both autosomal and sex-linked additive effects to empirical data from natural populations. Roulin *et al*. (2010) estimated autosomal and sex-linked heritabilities of a melanin-based plumage trait (i.e., the size of black spots located of the tip of feather of the ventral body side) in a wild population of Swiss barn owls (*Tyto alba*) and found that this trait was significantly influenced by sex-linked genes. Husby *et al*. (2013) estimated autosomal and sex-linked heritabilities (and additive genetic variances) of both morphological and (assumed) sexually selected traits for comparison in two long-term (pedigree) studies of a natural population of collared flycatchers (*Ficedula albicollis*) and a captive population of zebra finches (*Taeniopygia guttata*). Most traits in both species were not significantly influenced by sex-linked genes or showed low levels of sex-linked genetic variation. However, wing patch size in collared flycather (known to be under sexual selection) showed a higher level of sex-linked genetic variation.

The main focus in this article is to show how to explicitly model the additive effect of genes residing on the larger sex chromosome, that is, the X-chromosome which is found, for example, in most mammals and some insects (e.g., *Drosophila*) and the Z-chromosome found in birds, butterflies, moths, and some fishes (Russel 2006).

In this study, a simulation study is conducted for the barn owl study system to assess the identifiable properties of the model assuming both autosomal and sex-linked effects and to evaluate the consequences of using a model which only assumes autosomal inheritance when the trait under study is actually influenced by sex-chromosomal genes. We also present a detailed description on how to obtain the relevant precision matrices (inverse covariance matrices) required to explicitly account for and model sex-linked additive effects and set up an extended animal model. The objective is to provide a consistent framework allowing for estimation of both autosomal and sex-linked additive genetic effects using an animal model.

The methodology presented is also illustrated by analyzing the same melanin-based trait as in Roulin *et al*. (2010). Our approach do, however, avoid the numerical problems in inverting the precision matrix accounting for sex-linked additive genetic effects that were reported by Roulin *et al*. (2010), resulting in more precise estimates.

All inferences in this study are carried out using Bayesian methods. One of the main advantages of Bayesian methods compared with the more traditional REML methods is the more accurate representation of uncertainty in parameter and random variables estimates. Bayesian methods allow uncertainty to propagate through the model such that all available information is contained in the posterior distribution of the parameter and random variables in question. Although well established in the field of animal breeding (e.g., Sorensen and Gianola 2002), the use of Bayesian methods to tackle evolutionary questions has only recently been introduced (Kruuk *et al*. 2008; O'Hara *et al*. 2008; Ovaskainen *et al*. 2008; Hadfield 2010; Steinsland and Jensen 2010; Holand *et al*. 2013). We follow Holand *et al*. (2013), and use the Bayesian approximation methodology integrated nested Laplace approximations (INLA) introduced by Rue *et al*. (2009).

## Materials and Methods

### Field data

We use field data from a wild population of Swiss barn owls, a medium-sized nocturnal bird, in western Switzerland. In this study area covering 190 km^2^, 20–80 pairs of barn owls breed each year in 110 nest boxes put up in barns. We consider the plumage trait diameter of black eumelanic spots found on the tip of feathers on the owls' ventral side. The data in our study were recorded in the period between 1996 and 2007.

The number and size of eumelanic spots varies both within and among populations, and also within families (Roulin 2004). Spot size a sexually dimorphic trait, where females display on average larger black spots than males (females; mean = 13.13 mm [SD = 3.40], males; mean = 9.36 mm [SD = 3.96]). In this Swiss population, spot diameter has been shown to harbor a high heritability (*h*^2^ = 0.61) with some variation explained by genes on the Z-chromosome (Roulin *et al*. 2010). Furthermore, it has been shown that females, but not males, are positively selected for large spots (Roulin *et al*. 2010).

As extra-pair paternity is rare in the barn owls (Roulin *et al*. 2004), a pedigree was constructed by assuming that the social parents are the biological parents. Sex of nestlings was found using sex-specific molecular markers typed in blood cell DNA, and from the presence of a brood patch in breeding females (Roulin *et al*. 1999).

The pedigree consists of *N*_*p*_ = 2999 barn owls, with 1550 females and 1449 males. Plumage spots are expressed already at the nestling stage, and spot diameter is measured for most individuals in the pedigree (*N*_*d*_ = 2543, 1333 females and 1210 males). The spot diameter data are standardized to have mean 0 and variance 1. Further, sex and hatch year is available for all individuals in the pedigree and has been found to be important for both variation in and selection on plumage spot diameter (Roulin *et al*. 2010). The plumage spot diameter is approximately Gaussian distributed (see Fig. S1). Mean spot diameter for both sexes and for females and males separately for each cohort (i.e., hatch year) is given in Fig. S2 and suggests changes in spot diameter over the study period. For a more thorough description of the fieldwork and methods, study area, and genetic analyses, see, for example, Roulin *et al*. (2010) and references therein.

There are some differences in the dataset used in this study compared with Roulin *et al*. (2010), and the reason for using slightly different datasets is further explained in the Discussion. First, Roulin *et al*. (2010) used a pedigree consisting of *N*_*p*_ = 3264 individuals captured between 1987 and 2007, and this is the same pedigree as in our study except that the parents of the individuals with hatch year 1996 was included (i.e., they were the "founders") in their study. These individuals were also included in the dataset used in Roulin *et al*. (2010), giving *N*_*d*_ = 2711 individuals. Second, the dataset in Roulin *et al*. (2010) included all individuals alive in 2007, whereas in the dataset used in our study all individuals in the pedigree with hatch year before 2006 were taken out. Furthermore, in this study, the phenotypic trait was standardized to have mean zero and variance 1. In contrast, Roulin *et al*. (2010) standardized the data within each sex, which resulted in phenotypic data with mean zero and variance 1 within each sex.

### Animal model

To introduce the animal models, we review models presented in, for example, Lynch and Walsh (1998), Sorensen and Gianola (2002) and Kruuk (2004). We first introduce a Gaussian version of the animal model for only autosomal loci. The model is further extended to also include sex-linked inheritance.

An animal model for autosomal inheritance (AI) is a (generalized) linear mixed model where the observed trait values *y*_*i*_, *i* = 1,…,*N*_*d*_ are given by:


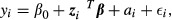
(1)

where *β*_0_ is an intercept, ***β*** = 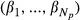 are referred to as fixed effects that account for group-specific effects such as, for example, sex and hatch year (although in theory all Bayesian parameters are random) and 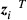 is a known incidence vector. The *a*_*i*_'s are individual additive genetic effects and are genetically linked random effects also known as breeding values. 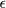
_*i*_ is individual *i*'s residual effect, and is an unstructured Gaussian random effect, often called the environmental effect in quantitative genetics. The parameters ***β***, 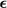, and ***a*** are assigned independent Gaussian priors, 
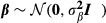
, the residual effects 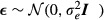, where ***I*** is the identity matrix and 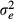 is generally referred to as the environmental variance. The additive genetic effects of the autosomal loci are for the population, 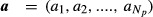, assumed to have a covariance matrix 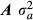, with a dependency structure corresponding to the pedigree 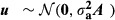, where ***A*** is the relationship matrix whose elements are twice the coefficient of co-ancestries between relatives for autosomal loci, and 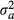 is the additive genetic variance in the base population (see e.g., Lynch and Walsh 1998; Sorensen and Gianola 2002). According to ***A***, an individual receives half of its autosomal genes from each of its parents irrespective of sex (Quaas 1976), and 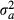 is an estimate for additive genetic variance for autosomal loci. Hence, the model in eqn (1) models the additive effects of genes located on autosomes.

To include the additive genetic effects of the sex chromosomes, we model the additive genetic effect of genes residing on the largest of the sex chromosomes, for birds the *Z*-chromosome, and assume the smallest chromosome (here *W*) is inert with respect to additive effects (Fernando and Grossman 1990; Ellegren 2007). The total additive genetic effect is then partitioned into the sum of additive effects due to autosomal genes and additive effects due to Z-linked genes. Statistically, it is straightforward to include a new random variable in the animal model, such as the Z-linked additive genetic effect, given its corresponding covariance structure is available. We can extend the AI animal model in eqn (1) to an autosomal and Z-linked inheritance (AZI) animal model accounting for both autosomal and sex-linked additive genetic effects:


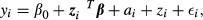
(2)

where *z*_*i*_ is the individual *i*'s additive genetic effects due to genes on the sex chromosome. The additive genetic effects of the *Z*-chromosome for the population 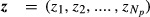 are assumed to have a covariance matrix 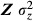, with a dependency structure corresponding to the pedigree and the sex of individuals in the pedigree. It is given a Gaussian prior 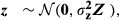 where ***Z*** is a matrix whose elements are functions of the coefficient of co-ancestries between relatives for the Z-chromosomal loci, and 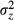 is the variance of additive genetic effects for sex-chromosomal genes for the homogametic sex, here males, in the base population (Fernando and Grossman 1990).

The underlying theory for computation of ***Z*** rests on some assumptions. The population is assumed to be in gametic equilibrium, the additive genetic effect for the same allele is assumed to be equal for males and females (no dosage compensation Ellegren *et al*. 2007b; Itoh *et al*. 2007), and allelic frequencies are equal in the two sexes. ***Z*** differs from ***A*** because the sex-linked genes are transmitted in a different pattern than the autosomal genes. A female (the heterogametic sex, here ZW) receives all of her Z-linked genes *z* from her paternal parent (the homogametic parent, here ZZ) and no Z-linked genes from the maternal parent (the heterogametic parent, here ZW), as mothers pass on their W-chromosome to daughters. On the other hand, a male will receive *z*_*m*_ from his maternal parent and *z*_*p*_ from his paternal parent, *z* = *z*_*m*_ + *z*_*p*_. Thus, the additive Z-linked genetic variance for noninbred males (homogametic sex) is 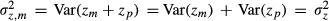, while for noninbred females (heterogametic sex), 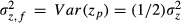. Hence, for a noninbred population with an 1:1 sex ratio, the total variance in the population due to Z-linked inheritance is 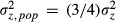.

Throughout, ***A*** will be referred to as the autosomal relationship matrix, and ***Z*** will be referred to as the Z-linked relationship matrix. We assigned inverse Gamma priors *σ*^2^*∼invGamma(a*, b*), where a* = 1 and b* = 0.001 to variance parameters 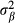, 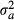, 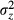, and 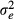.

### Modeling Z-linked inheritance – INLA and computational issues

We use integrated nested Laplace approximations (INLA) to estimate variances 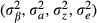, individual breeding values (*a*_*i*_, *z*_*i*_) and DIC from AI animal models (eqn (1)) and AZI animal models (eqn 2). INLA is a fast and deterministic nonsampling-based approach to Bayesian inference available for latent Gaussian Markov random field (GMRF) models (Rue *et al*. 2009). It has been shown that the AI animal model falls within the class of GMRF models, and INLA can be used as inference method (Steinsland and Jensen 2010; Holand *et al*. 2013).

For INLA methodology to work efficiently, the latent Gaussian model has to satisfy some properties. The latent Gaussian field ***x***, generally of large dimension, must admit conditional independence properties. Thus, the latent Gaussian field is a Gaussian Markov random field (GMRF) with a sparse precision matrix (inverse of covariance matrix) ***Q*** (Rue and Held 2005), as the efficiency of INLA relies on efficient algorithms for sparse matrices. Due to the use of a numerical integration scheme and optimization methods in INLA, it needs to integrate over the non-Gaussian hyper-parameter space, and therefore, the dimension of non-Gaussian hyperparameters ***θ*** cannot be too large, say ≤14. In addition, the likelihood for each observation *y*_*i*_ depends on the latent Gaussian field only through the linear predictor *η*_*i*_ = *g*(*μ*_*i*_), where *g*(·) is a known link function and *μ*_*i*_ = *E*(*y*_*i*_|***x***, ***θ***), that is, *π*(*y*_*i*_|***x***, ***θ***) = *π*(*y*_*i*_|*η*_*i*_, ***θ***).

The AI (eqn 1) and AZI (eqn 2) animal model can be formulated in the INLA framework with a Gaussian likelihood 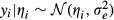 and an identity link function, *η*_*i*_ = *μ*_*i*_, where *η*_*i*_ is the linear predictor. The linear predictor in the AI model can be written as:


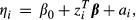
(3)

and the linear predictor in the AZI model as:


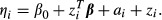
(4)

It is shown (Henderson 1976; Quaas 1976; Steinsland and Jensen 2010; Holand *et al*. 2013) that the inverse of the autosomal relationship matrix ***A***^−1^ is a sparse matrix, which can be calculated from the pedigree. Further, the inverse of the sex-linked relationship matrix ***Z***^−1^ is also a sparse matrix, which can be calculated from the pedigree and sex information (Fernando and Grossman 1990). The autosomal and Z-linked genes are on different chromosomes; therefore, ***a*** and ***z*** are assumed independent, and their joint precision matrix is also sparse. These two precision matrices are easily fitted into the INLA framework. The latent field ***x*** = (***β***, ***a***, ***z***) therefore admits conditional independence properties, such that ***x*** is a GMRF, where the precision matrices for the latent field are sparse. As the number of non-Gaussian hyperparameters 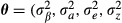) is small, and the likelihood of each observed trait, *y*_*i*_, depends on the latent field only through the linear predictor *η*_*i*_, the requirements for INLA are fulfilled also for the AZI animal model.

The R software (R Development Core Team 2013) were used in our study. The R–INLA package (available at: http://www.r-inla.org) makes inference from GRMF models using the INLA methodology. Further, the R-package AnimalINLA includes functionality for calculating *A*^−1^ and *Z*^−1^, and can be downloaded at: http://www.r-inla.org. The details of the procedure to efficiently compute *Z*^−1^ directly from pedigree and sex information are given in the Appendix S1.

### Model comparison

Model comparisons in both the simulation study and in the barn owl case study are carried out using the deviance information criterion (DIC), which is a measure of complexity and fit (Spiegelhalter *et al*. 2002). The model with the smallest DIC is considered the best model, and according to Spiegelhalter *et al*. (2002), differences in DIC, ΔDIC, of more than 10 should definitely rule out the model with the higher DIC.

In Holand *et al*. (2013), a simulation-based test of the ability of the difference in DIC to chose between animal models with and without genetic effects was presented. Here, we followed the same ideas and conducted a simulation-based hypothesis test to test whether Z-linked inheritance can be identified using ΔDIC. Under the null hypothesis, *H*_0_, the AI animal model is true. Under the alternative hypothesis, *H*_1_, the AZI animal model is true. To estimate the probability of type-I error (reject *H*_0_ when it is true), we sample *S* datasets from the AI animal model. For each of these *s* = 1,…, *S* datasets, we fit both an AI model and an AZI animal model and calculate the difference in DIC, ΔDIC_*s*_ = DIC(AI)_*s*_−DIC(AZI)_*s*_. The obtained *S* values of ΔDIC are then be used as an approximation to the sampling distribution of ΔDIC under the null hypothesis. As we reject the null hypothesis for ΔDIC > 10, the proportion of ΔDIC > 10 is an estimate for the probability of type-I error.

We also find the power (the probability of rejecting *H*_0_ when *H*_1_ is true) of the test for some chosen values of 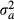, 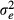, and 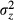. For each parameter set, we sample *S* datasets from the AZI animal model, fit both an AI animal model and an AZI animal model, and calculate ΔDIC for these models. The proportion of the *S* ΔDIC values larger than our chosen limit of ΔDIC = 10 is an estimate of power when there are some sex-linked genetic effects.

### Simulation study

The aim of the simulation study was threefold: (1) to assess the impact of ignoring Z-linked inheritance on estimated variance components when sex-linked inheritance is present, (2) to evaluate the ability of ΔDIC to choose between models with and without sex-linked genetic effects, and (3) to evaluate bias and coverage of variance parameters for the two animal models (AI and AZI).

For the simulation study, we use the barn owl pedigree presented in Materials and Methods, Field data, and we also impose the same missing data structure in the simulated dataset as in the barn owl dataset. Therefore, we can also validate whether the barn owl study system is suitable for identifying Z-linked inheritance. We sample data from the AZI animal model defined in eqn (2) for the pedigree described in Material and Methods, Animal model for chosen sets of parameters. These parameters are chosen as follows: we simulate approximately standard Gaussian datasets by setting *β* = 0 and the total variance 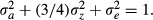 Further, the heritability


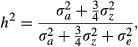
(5)

is fixed to *h*^2^ = 0.6, hence 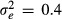 and 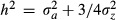. By choosing 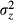
={0, 0.1, 0.2, 0.3, 0.4, 0.5, 0.6, 0.7, 0.8}, the corresponding values for autosomal variance are 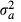
={0.6, 0.525, 0.450, 0.375, 0.3, 0.225, 0.150, 0.075, 0}. These parameter sets range from only autosomal inheritance 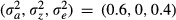, that is, the AI animal model, to only sex-linked inheritance 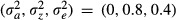.

One thousand replicated datasets (*S* = 1000) were simulated for each of these nine parameter sets. Each dataset was fitted to both the AI animal model in eqn (1) and the AZI animal model in eqn (2). Posterior mean and 95% credible intervals for variance parameters as well as DIC were calculated for each model, and ΔDIC for each pair of models.

To summarize the simulation results, we also calculated the bias and coverage for each parameter set and each model. Bias is a measure of the accuracy of an estimator 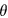 of a parameter *θ*, and defined as 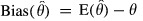, where 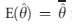 is the average of the 1000 estimated variance parameters and *θ* is the parameter value used in the simulations. Further, we use the coverage to assess the precision of the estimator, which is the proportion of times the true parameter *θ* falls within the 95% credible interval of 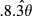, calculated as number of times the parameter value used in the simulations are either larger or smaller than the estimated lower (0.025%) and upper (0.975%) quantiles out of the 1000 simulations.

### Model for field data

We analyze the data in Materials and Methods, Field data, where the plumage spot diameter is assumed to have a Gaussian likelihood using the AZI animal models defined in Materials and Methods, Animal model. The inference is carried out using INLA described in Materials and Methods, Modeling Z-linked inheritance – INLA and computational issues.

First, we do a model comparison using DIC to choose which fixed effects (sex and hatch year) and random effects (autosomal or Z-linked additive genetic effect) to include in our model.

We started with a full model: *y*_*i*_ = *β*_0_ + *β*_sex(i)_ + *β*_hatch year(i)_ + *a*_*i*_ + *z*_*i*_ + *ε*_*i*_ and removed one variable at a time in a stepwise manner. In each step, all nested models are examined, where we only report the one with the lowest DIC (i.e., the best at each step). For comparison, we also fitted the best model without sex-linked variance. For all fixed-effects parameters *β*, we use the prior for the covariates 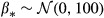.

To examine whether any evolutionary trends in posterior mean of mean additive genetic effects in spot diameter had occurred over the study period, we find linear combinations of both autosomal and ***Z***-linked mean additive genetic effects for each hatch year (i.e., cohort); 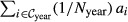 and 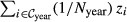, where *N*_year_ is the number of individuals with a given hatch year and summing over all these individuals (Sorensen *et al*. 1994). For further details, see Holand *et al*. (2013).

## Results

### Simulation study

**Figure 1:**
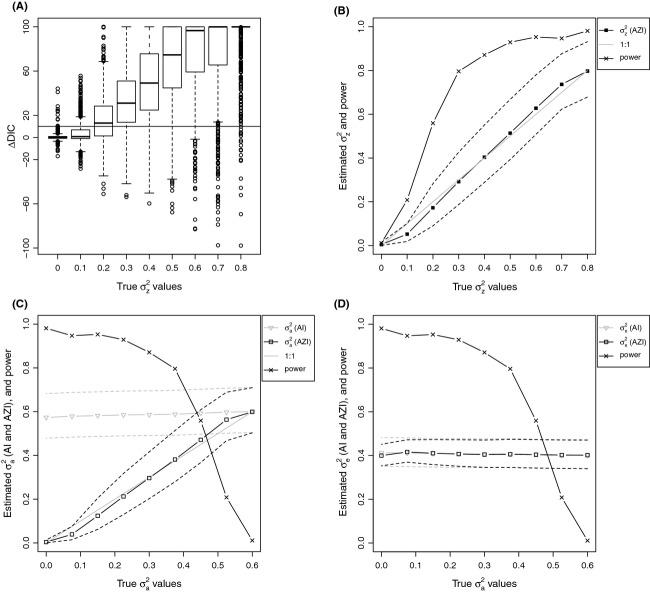
Results from simulation studies showing performance of animal models for estimating Z-linked additive genetic effects. (A) Boxplot of simulated values of ΔDIC (limit ΔDIC = 10 indicated as a horizontal solid line) against the value of 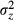 (AZI) used in the simulations (together with 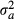 and 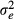). (B) Posterior mean (filled squares/solid lines) with 95% credible interval (dashed line) for 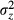 (AZI) from the simulation study (together with 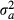 and 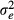), power of the model selection test using ΔDIC >10 as limit (x'es/solid line) estimated using the simulation approach, and a 1:1 function of true vs. estimated parameter values (gray line). (C) Posterior mean (open triangles/solid line) for 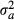 (AI) (gray) with 95% credible interval (dashed lines) and for 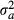 (AZI) (open squares, black) with 95% credible interval (dashed lines) from the simulation study (together with 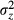 and 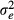), power of the model selection test, and 1:1 function as described in panel (A). (D) Posterior mean (solid lines) for 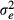 (AI) (gray) with 95% credible interval (dashed lines) and for 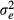 (AZI) (black) with 95% credible interval (dashed lines) from the simulation study against the value of 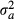 used in the simulations (together with 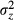) and power function as described in (A).

To evaluate the ability of ΔDIC to identify sex-linked inheritance, we consider the results of ΔDIC from the simulation study, see Fig. 1 panel A where boxplots of obtained ΔDIC for different values of 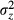 are plotted. Of the *S* = 1000 datasets simulated from a model with only autosomal inheritance (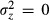), we find that only eleven datasets have ΔDIC > 10, and hence, we have an estimated probability of type-I error (i.e., significance level) of only 0.011. The power of the test (i.e., the probability of correctly rejecting *H*_0_ when there is sex-linked inheritance) can be found in Fig. 1 panel B as a function of 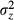. We find that for 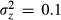, the power is only 0.23, but it increases fast and is already 0.53 for 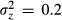 and 0.73 for 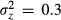. Hence, for the barn owl system, we are able to detect sex-linked inheritance if there is a relatively substantial amount of sex-linked effects.

To evaluate the consequences of not including sex-linked inheritance in the model when it is present, we consider Fig. 1 panel C where the estimated values of 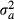 are plotted when fitting an AI animal model against the true value (gray lines). We find that regardless of the true value of 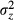, the estimate is close to the total amount of additive variance (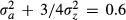). This results in large bias and low coverage for 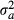 when fitting an AI animal model when sex-linked inheritance is present, see Table S1. From Fig. 1 panel D, we see that not including sex-linked inheritance has very little effect on the estimated values of 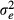.

From Table S1 we see that, when fitting the AZI animal model, the bias is small and coverage is good except for small values of the additive variances 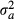 and 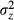. This is known to be due to prior sensitivity (see Holand *et al*. 2013). When models are fitted to a dataset without sex-linked inheritance (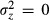 and 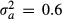 in out simulation study), we see from Fig. 1 panel B, C, and D that the AZI estimates perform slightly worse than the AI estimates in terms of larger credible intervals.

### Plumage spot diameter in barn owls

The results of the model comparison where different models were fitted to the spot diameter data and compared in terms of their DIC values are listed in Table1. The difference in DIC values for the model, including both autosomal and Z-linked effects versus the model accounting only for autosomal effects, was 180 in favor of the model which explicitly models Z-linked additive genetic effects. Difference in DIC thus greatly exceeds the chosen limit of 10 and decisively indicates that spot diameter is partially influenced by variation in Z-linked genes. Furthermore, the overall best model includes sex as fixed effect in addition to both autosomal and Z-linked additive genetic effects.

**Table 1:** Different model specifications explaining variance in spot diameter of Swiss barn owls and the corresponding deviance information criteria (DIC)

Model	DIC
Sex + hatch year + autosomal effect + Z-linked effect	5050
**Sex + autosomal effect + Z-linked effect**	**4757**
Sex + autosomal effect	4937
Autosomal effect	5898

The best model is given in bold.

The estimated posterior mean and 95% credible interval for the additive genetic variances were 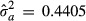 (95% CI: 0.3603 to 0.5336), 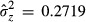 (95% CI: 0.1833 to 0.3880), and 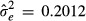 (95% CI: 0.1639 to 0.2439). The parameter 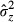 is the Z-linked additive genetic variance for noninbred males. Thus, for females, we have 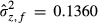 (95% CI: 0.0917 to 0.1940).

In comparison, a model including sex and only autosomal additive genetic effect yields additive genetic variance 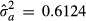 (95% CI: 0.5392 to 0.6936) and 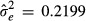 (95% CI: 0.1799 to 0.2650).

**Figure 2:**
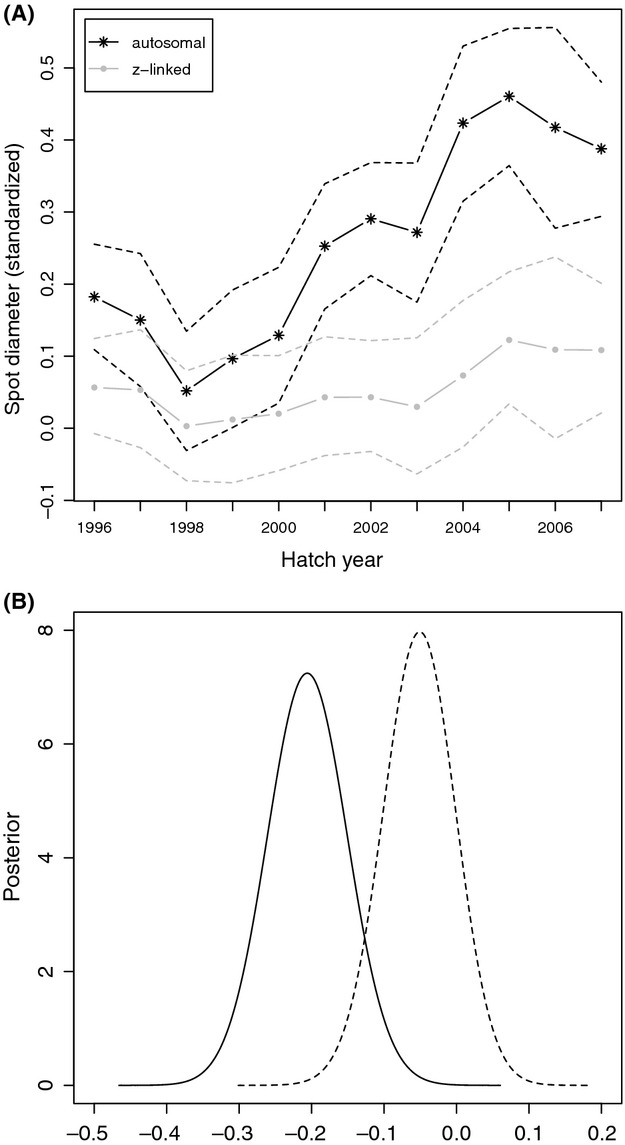
(A) Posterior mean of mean additive genetic effect for spot diameter of all individuals in the pedigree for each cohort (i.e., hatch year) 1996–2007 for autosomal loci (black) and Z-linked loci (gray) (solid lines) with 95% credible intervals (dashed lines). The mean spot diameter was standardized to have mean 0 and variance 1. (B) Posterior of difference between cohorts 1996 and 2007 in mean additive genetic effects for autosomal loci (solid lines) and Z-linked loci (dashed lines) for spot diameter in Swiss barn owls.

The linear combinations of posterior mean of mean additive genetic effects across cohorts suggest for spot diameter that there was an increase in additive genetic effect for autosomal loci, but no increase in the additive genetic effect for Z-linked loci (Fig. 2). To test this, we investigated whether the difference between cohorts 1996 and 2007 in posterior mean additive genetic effects was significant for either autosomal and Z-linked loci: 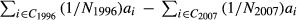 and 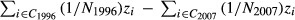, respectively. The difference between additive genetic effects for cohorts 1996 and 2007 was significant for autosomal loci, with mean difference −0.206 (SD = 0.055) and 95% CI (−0.313,−0.097). In contrast, the difference was not significant for Z-linked loci; mean difference −0.052 (SD = 0.050) and 95% CI (−0.152,0.046). The posterior marginals of the difference for autosomal and Z-linked loci are given in Fig. 2.

## Discussion

From the simulation results, we see that although we have modeled only autosomal inheritance, in the presence of Z-linked inheritance in a trait, the total amount of additive genetic variance is correct. This is apparently because all the additive genetic variance, including the part due to genes on the Z-chromosome, is picked up by the estimated autosomal additive genetic variance. Hence, using an AI animal model when an AIZ model is true gives an estimate of autosomal additive genetic variance, which corresponds to the total amount of additive genetic effects in the AZI model. Incorrect predictions of responses to selection may, however, be one of the consequences of not modeling Z-linked inheritance when it exists. Results from the simulation study therefore clearly illustrate the importance of specifying the correct model in the presence of Z-linked inheritance. Further, the simulation study also demonstrates that for our barn owl study system (i.e., this type of pedigree and missing data structure), difference in DIC between AI and AIZ models is a good measure for model choice. Study systems that show low support for sex-linked effects, for example, Husby *et al*. (2013), would benefit from a simulation study to explore the system's ability to separate autosomal and sex-linked additive genetic effects. In our simulation study, we used 1000 datasets; however, more datasets might be needed if a more robust estimation of a specific power is desired.

The analysis of spot diameter in the empirical barn owl dataset showed that spot diameter is clearly influenced by both autosomal and Z-linked additive genetic effects. The results show that spot diameter is under strong genetic influence, and genes on the Z-chromosome contribute a substantial amount to the total phenotypic variation.

According to theory on effects of selection when it mainly acts on the heterogametic sex, for example, the females in birds and males in mammals, we expect to see a change in mean additive genetic effects mainly in the genes found in the autosomes (Charlesworth *et al*. 1987). This is in accordance with the results found in this study, where the changes in posterior mean of mean additive genetic effects of spot diameter across cohorts suggest an increase in autosomal additive genetic effects over the study period (Fig. 2A). This is supported by the significant difference between cohort 1996 and 2007 found for the autosomal additive genetic effects, whereas there was no significant change across cohorts for the Z-linked additive genetic effects (Fig. 2B). However, it is difficult to determine whether the observed change in mean breeding values is due to an evolutionary response to selection on spot diameter or random genetic drift, as genetic drift may cause independent fluctuations in breeding values across generations (Hadfield *et al*. 2010). In any case, the result that the genetic changes mainly occurred on the autosomes corresponds with other studies of birds, suggesting that most of sexually antagonistic genes beneficial for females are located on the autosomes (Ellegren and Parsch 2007; Mank and Ellegren 2009).

Another possible explanation to the small change in breeding values over the cohorts for Z-linked genes is that the spot diameter itself is not under selection, but rather another trait that is genetically correlated with spot diameter on the autosomal chromosomes is under selection. This is in accordance with findings in Roulin and Ducrest (2011), which showed that spot size displayed by mothers is correlated with offspring quality measures including parasite resistance, resistance to oxidative stress, and an increase in corticosterone levels, appetite, and the ability to withstand lack of food.

Sex-specific selection is the process in which selection is favoring different optimal character states in the two sexes, a mode of selection that recently has received much attention by evolutionary biologists (see e.g., Lande 1980; Foerster *et al*. 2007; Cox and Calsbeek 2009; Mills *et al*. 2012; Stearns *et al*. 2012). Modeling sex-linked genetic variance and performing a simulation study to explore the strength of the study system to identify sex-linked genetic variance are especially important when working with sexual conflict and sex-specific selection. The covariance between a given trait and selection can be positive for males and negative for females, or the other way around. This type of selection may, for example, occur because the two sexes have differing roles in reproduction, leading to different phenotypic optima in females and males. The study of sex-specific selection is interesting because this pattern of selection may account for the evolutionary stability of sexual dimorphism, it may also explain why genetic variation is not eroded, and it provides interesting implications into the understanding of intralocus genetic conflict (Bonduriansky and Chenoweth 2009). This type of conflict results from the fact that different alleles are favored in the two sexes, which can result in intricate phenomena such as sex ratio bias (Blackburn *et al*. 2010).

Our models assume that the additive genetic effects are the same in both sexes, that is, the intersex genetic correlation is one. As the spot diameter in the Swiss barn owls is a sexually dimorphic trait and has been shown to be subject to sex-specific selection (Roulin *et al*. 2010), this assumption could be violated, which might results in some bias in the estimated autosomal and sex-linked additive genetic variances. To explore this, a model treating the spot diameter in males and females as two different traits that are genetically correlated has to be fitted. This is, however, outside the scope of this study, but studies of different species suggest that intersex correlations of homologous morphological traits often are close to one (Jensen *et al*. 2008; Kruuk *et al*. 2008).

The animal model we have used (implicitly) assumes that both autosomal and sex-linked genetic effects are additive, that there is no sex chromosome dosage compensation, and that missing observations are missing at random. Nonadditive effects such as dominance and epistasis are known to be hard to identify from nonexperimental study systems (Lynch and Walsh 1998), and it is outside the scope of this work to our extend model to account for these effects. Other studies have suggested that sexually antagonistic genes often are dominant (Ellegren and Parsch 2007; Mank and Ellegren 2009). However, the importance of nonadditive genetic effects is arguable as some studies suggest both that dominance and epistasis effects may contribute little to the phenotypic variance (Merilä *et al*. 2001; Visscher *et al*. 2007; Crow 2010, but see e.g. Carlborg and Haley 2004).

Hence, dominance and epistasis may not affect our results considerably. The assumption of no sex chromosome dosage compensation seems to be a good assumption as an overall dosage compensation has not been found in birds (Ellegren *et al*. 2007a; Itoh *et al*. 2007). The assumption that missing observations of the trait of interest are missing at random is further explored in Steinsland *et al*. (2014), where it is concluded that for this system, the assumption does not influence the variance estimates to any large extent.

Both the model and the data used in Roulin *et al*. (2010) and our study are slightly different. Therefore, we do not expect results in these two papers to coincide exactly. However, the results found in the two studies are essentially identical when it comes to sex-linked variances. In Roulin *et al*. (2010), the Z-linked additive genetic variance 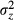 was reported for females (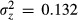), while it is reported for males here. Consequently, if we compare (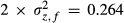) with 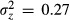, we see that the additive sex-linked variance is very similar. Furthermore, the trends in additive genetic effects are similar in Roulin *et al*. (2010) and in the current paper. Posterior distributions of mean difference in mean breeding values were, however, not exactly equal in the two studies, probably as a consequence of different models and data used. In Roulin *et al*. (2010), phenotypes of more owls were included, data were standardized within each sex (see also Steinsland *et al*. 2014), and hatch year but not sex was included as fixed effect in their animal model. It should be noted that standardizing the phenotypes within each sex forces the variance within each sex to be equal, while an animal model with sex-linked effects implicitly assumes that males have larger variance than females. Hence, it is inconsistent to do sex-specific standardization prior to applying an animal model with sex-linked inheritance. Finally, different methods for computing ***Z***^−1^ were used. In Roulin et al. (2010), the software Mendel (Lange *et al*. 2001) was used to compute ***Z***, and MATLAB to invert this matrix to obtain ***Z***^−1^. Numerical problems with this approach were reported.

## Conclusion

We have in this study introduced a methodology for estimation and testing identifiability issues regarding sex-linked additive genetic effects and discussed consequences of not modeling this variance when it is present. Through a simulation study, we have shown that for a real wild population system (with a given pedigree, missing data structure, and sex distribution) that both autosomal and sex-linked effects can be estimated, these effects can be distinguished (i.e., they are identifiable), and difference in DIC between animal models with only autosomal inheritance and both autosomal and sex-linked inheritance can be used to test whether sex-linked inheritance is present. Using an animal model with only autosomal inheritance when sex-linked inheritance is present results in inflated estimates of the autosomal additive variance, as it also includes the sex-linked variance of the population. This might give misleading interpretations, especially when response to sex-specific selection is studied, as the heterogametic sex for instance will have a slower response to selection than the homogametic sex for genes on the sex chromosome when alleles have largely dominant effects (Charlesworth *et al*. 1987). We are not able to obtain any knowledge about potential sex-linked inheritance from a model assuming only autosomal inheritance. On the other hand, fitting an animal model with both autosomal and sex-linked effects to a system where no sex-linked effects are present, performs approximately equally well as the model that (correctly) assumes only autosomal inheritance. We therefore recommend that animal models including both autosomal and sex-linked effects are used, or at least tested.

In our study of plumage spot diameter in a Swiss barn owl population, we found that sex-linked effects account for a substantial proportion of the phenotypic variance. Earlier results indicated that this trait is under sex-specific selection (Roulin *et al*. 2010).

## Supporting Information

**Figure S1.** Histogram over spot diameter for Swiss barn owls.

**Figure S2.** Mean phenotypic spot diameter over cohorts.

**Table S1.** Simulation results in terms of bias and coverage.

**Appendix S1.** Calculation of the inverse Z-linked relationship matrix.
