# Supplementary material for: On estimation and identifiability issues of sex-linked inheritance with a case study of pigmentation in Swiss barn owl (Tyto alba)
Source: Ecol Evol. 2014 Mar 29;4(9):1555–66. doi: 10.1002/ece3.1032 (PMC4063458; doi:10.1002/ece3.1032)
Supplement: Supplementary file 1 [file ece30004-1555-SD1.pdf]

Supporting Information

On estimation and identifiability issues of sex-linked inheritance with a case study of pigmentation in Swiss barn owl (*Tyto alba*)

Camilla Thorrud Larsen[*,***], Anna Marie Holand[§,1],
Henrik Jensen[**], Ingelin Steinsland[§,§§§], Alexandre Roulin[§§,§§§]

[*]Current address: Department of Electric Power Engineering, NTNU, NO-7491 Trondheim, Norway, [**]Centre for Biodiversity Dynamics, Department of Biology, NTNU, NO-7491 Trondheim, Norway, [***]Department of Mathematical Sciences, NTNU, NO-7491 Trondheim, Norway, [§]Centre for Biodiversity Dynamics, Department of Mathematical Sciences, NTNU, NO-7491 Trondheim, Norway, [§§]Department of Ecology and Evolution, 1015 Lausanne, University of Lausanne, Switzerland, [§§§]Senior authors

[1]Corresponding author: Anna Marie Holand, Centre for Biodiversity Dynamics, Department of Mathematical Sciences, Norwegian University of Science and Technology, NO-7491 Trondheim, Norway, Email:
anna.holand@math.ntnu.no

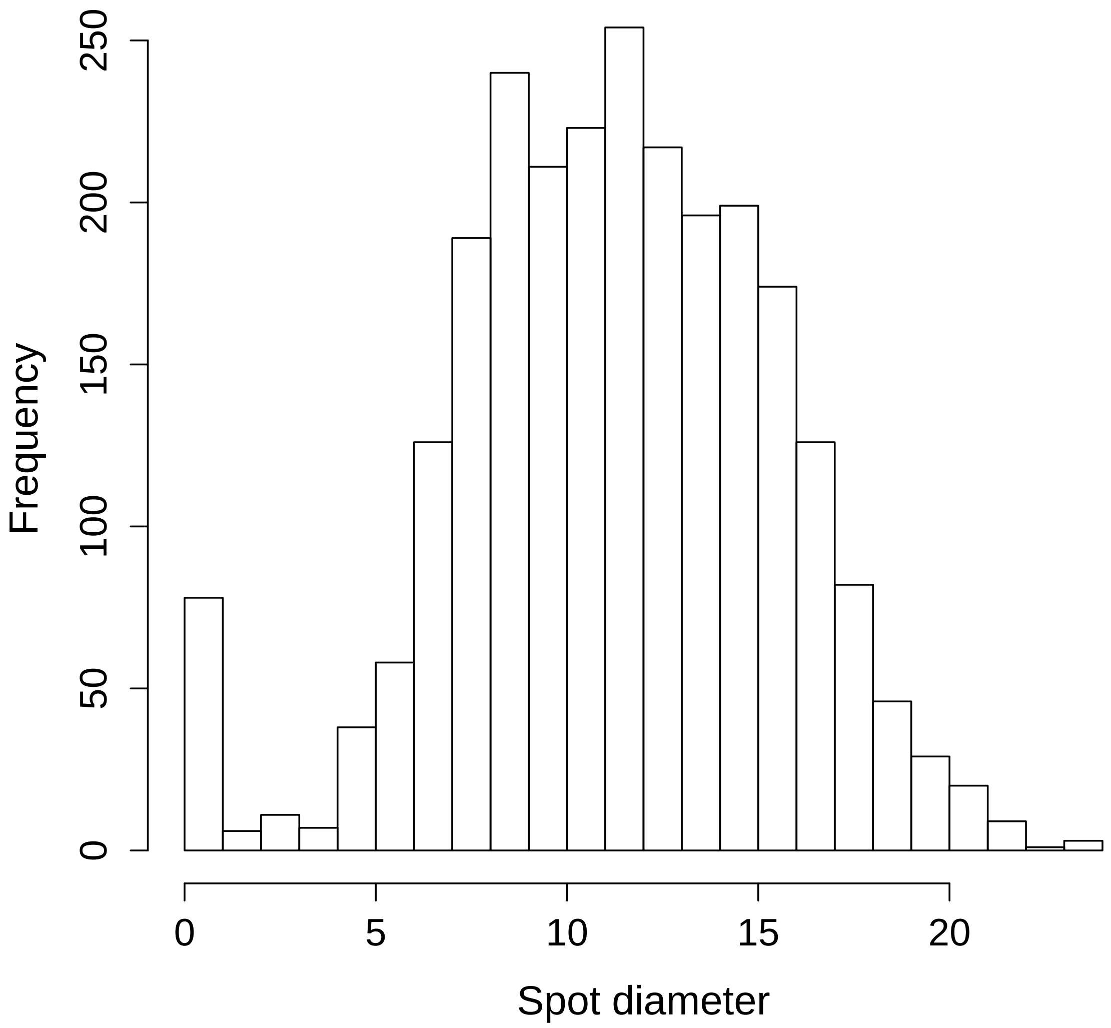

Figure S1 Histogram over spot diameter for Swiss barn owls. Histogram showing phenotypic spot diameter observations for Swiss barn owls, indicating Gaussian distribution.

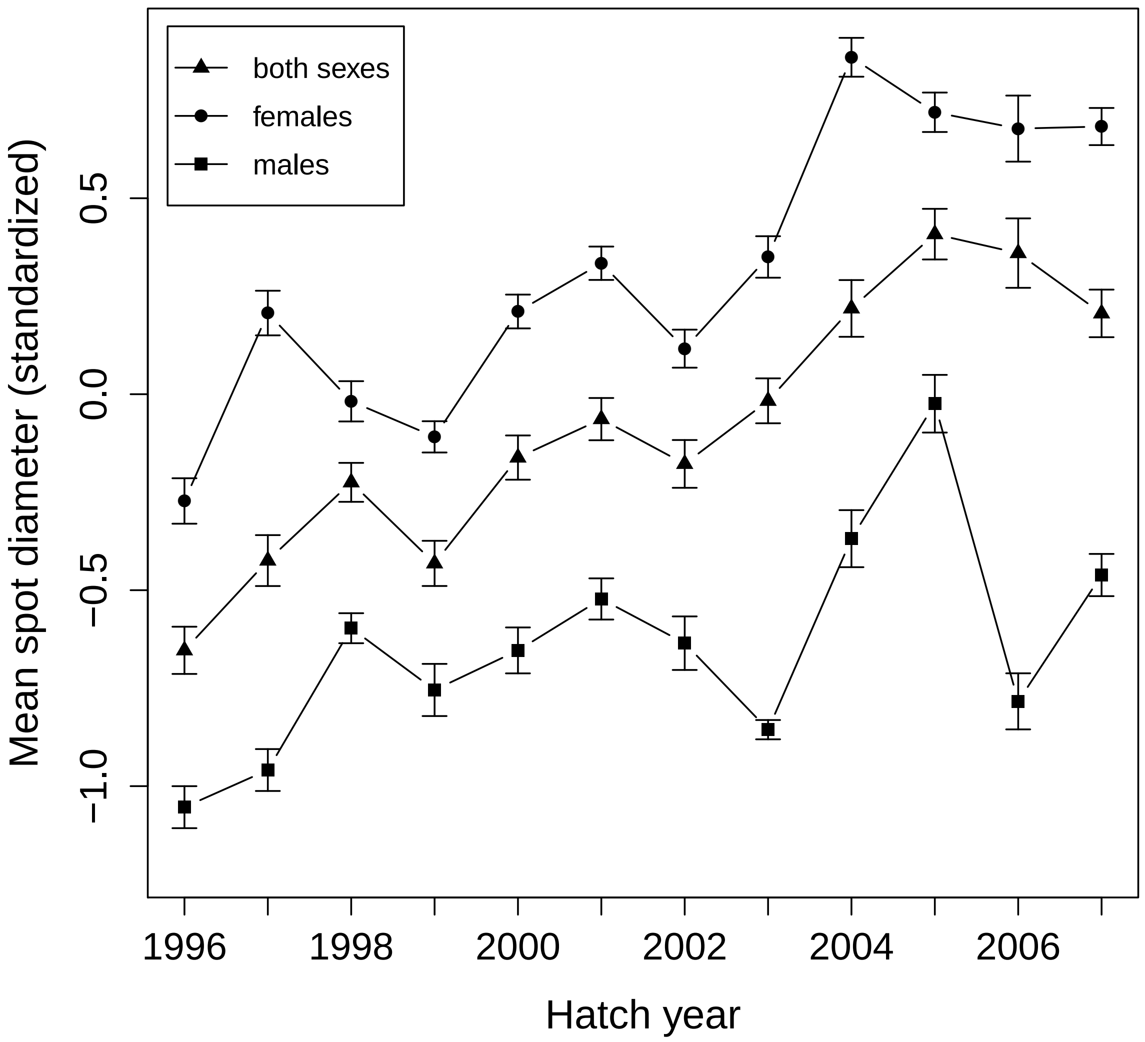

Figure S2 Mean phenotypic spot diameter over cohorts. Mean phenotypic spot diameter for females (dots), males (squares) and both sexes (triangles), with 95% confidence intervals, for cohorts (i.e. hatch years) 1996-2007. The mean spot diameter are standardized to have mean 0 and variance 1.

Appendix S1: inverse of the Z-linked relationship matrix

The non-zero structure of the inverse of Z-linked relationship matrix is found from pedigree and sex information, and the non-zero values can be calculated according to Fernando & Grossman (1990). The procedure returns the non-zero elements of $\boldsymbol{Z}^{-1}$ directly without computing $\boldsymbol{Z}$, which would be computational demanding and numerically unstable due to the large number of computations required. The underlying theory for computation of $\boldsymbol{Z}^{-1}$ rests on some assumptions. First, the additive effects of the same allele is assumed to be equal for males and females and there is no dosage compensation. Secondly, the population is assumed to be in gametic equilibrium (Fernando & Grossman, 1990).

The critical feature of the procedure presented in Fernando & Grossman (1990) is the recognition that the Z-linked relationship matrix $\boldsymbol{Z}$ can be expressed as

$$\boldsymbol{Z} = (\boldsymbol{I} - \boldsymbol{P})^{-1}\boldsymbol{V}(\boldsymbol{I} - \boldsymbol{P}')^{-1},$$

where a prime denotes the transposed and that its inverse is given by

$$\boldsymbol{Z}^{-1} = (\boldsymbol{I} - \boldsymbol{P}')\boldsymbol{V}^{-1}(\boldsymbol{I} - \boldsymbol{P}). \tag{1}$$

Here, $\boldsymbol{I}$ is the identity matrix and $\boldsymbol{P}$ is a matrix in which each row contains one or two non-zero elements if one or both parents are known, or all zeroes when both parents are unknown. The row for a female contains an element $\frac{1}{2}$ in the column corresponding to her paternal parent (half of fathers Z-linked genes are transmitted to his offspring, regardless of sex), while the column corresponding to her maternal parent is zero, since mothers transmit the w-chromosome to daughters. The row for a male individual also contains an element $\frac{1}{2}$ in the column corresponding to his paternal parent, and an element 1 in the column corresponding to his maternal parent (all of a mothers Z-linked genes are transmitted to sons). Thus, $\boldsymbol{P}$ describes the transmission of Z-linked genes through the sample/population. $\boldsymbol{V}$ is a diagonal matrix, whose elements depend on the sex of the corresponding individual and whether maternal and paternal parents are known or unknown (also, Z-chromosomal inbreeding coefficient of the paternal parent).This factorization is analogous the one for autosomal additive relationship matrix (Henderson et al.,

2000). Writing $\boldsymbol{Q} = (\boldsymbol{I} - \boldsymbol{P}')$, because $\boldsymbol{V}^{-1}$ is diagonal, equation 1 can be written as

$$\boldsymbol{Z}^{-1} = \sum_i \boldsymbol{q}_i \boldsymbol{q}_i' \frac{1}{v_i} \tag{2}$$

where $\boldsymbol{q}_i$ is the ith column of $\boldsymbol{Q}$ and v_i is diagonal element i of $\boldsymbol{V}$.

Following Fernando & Grossman (1990), the elements of $\boldsymbol{Q}$ and $\boldsymbol{V}$ can rapidly be acquired for any pedigree, provided sex is known for all individuals. The R-package `AnimalINLA` can be downloaded at $\mathtt{www.r - inla.org}$, it has a function which takes an arbitrary pedigree and sex information as input arguments, and returns the (non-zero) elements of $\boldsymbol{Z}^{-1}$.

To obtain $\boldsymbol{Z}^{-1}$ it is required that sex information is available for all individuals in the pedigree. Further, individuals must be numbered consecutively from 1 to N (N being the number of individuals in the pedigree) and ordered such that parents always precede their offspring.

The Z-chromosomal inbreeding coefficient is defined as the probability that two homologous alleles at a given Z-chromosomal locus are identical by descent (Malécot, 1969). The inbreeding coefficient is undefined for females, since only one allele resides at any Z-chromosomal locus. The inbreeding coefficient for males can be found from the Z-linked relationship matrix

$$f = z_{ii} - 1 \tag{3}$$

where z_{ii} is the ith diagonal element of the Z-linked relationship matrix. This requires calculation of Z, and is inefficient, especially for large pedigrees. The inbreeding coefficient f is required in the calculation of Z^{-1} to be able to calculate the inverse of Z directly. We now show how the Z-chromosomal inbreeding coefficient can be rapidly computed.

For individual i with maternal parent m and paternal parent p, the diagonal elements of $\boldsymbol{V}$ is computed as follows:

m and p are both known

$v_{ii} = \frac{1}{4}(1 - f)$, individual i is male or female

only m is known

$v_{ii} = \frac{1}{2}$, individual i is male or female

only p is known

$$v_{ii} = \begin{cases} \frac{1}{4}(1-f) & \text{if individual } i \text{ is female} \\ \frac{1}{4}(3-f) & \text{if individual } i \text{ is male} \end{cases}$$

m and p are both unknown

$$v_{ii} = \begin{cases} \frac{1}{2} & \text{if individual } i \text{ is female} \\ 1 & \text{if individual } i \text{ is male} \end{cases}$$

The algorithm returns the non-zero elements of the half-stored $\boldsymbol{Z}^{-1}$, as an array with three columns. The full matrix is obtained by symmetry. The first two columns contains the row- and column indexes, respectively, with the corresponding non-zero elements of $\boldsymbol{Z}^{-1}$ in the third column. This format is convenient due to the sparse structure of $\boldsymbol{Z}^{-1}$. For applications to species in which males are heterogametic (XY) and females homogametic (XX), such as most mammals, results should be interpreted accordingly. The following is saved in the ith round $(i = 1, ..., N)$

if individual i is a female with paternal parent p

$$(p, p, \tfrac{1}{4}d_i), \quad (i, i, d_i), \quad (p, i, -\tfrac{1}{2}d_i)$$

if p is unknown, omit elements involving p

if individual i is male with paternal parent p and maternal parent m

$$(p, p, \tfrac{1}{4}d_i), \quad (m, m, d_i), \quad (i, i, d_i)$$

$$(p, i, -\tfrac{1}{2}d_i), \quad (m, i, -d_i), \quad (\min(p, m), \max(p, m), \tfrac{1}{2}d_i)$$

if p is unknown, omit elements involving p

if m is unknown, omit elements involving m

Sort the array on column within rows, and sum over third row elements with equal row and column indexes.

References

Fernando, R.L. & Grossman, M. 1990. Genetic evaluation with autosomal and X-chromosomal inheritance. *Theoretical and applied genetics* **80**: 75–80.

Henderson, R., Diggle, P. & Dobson, A. 2000. Joint modeling of longitudinal measurements and event time data. *Biostatistics* **1**: 465–480.

Malécot, G. 1969. *The mathematics of heredity*. Freeman & Co.

Table S1 Simulation results in terms of bias and coverage. Simulation results for Gaussian simulated data simulated under model $y_i = \beta_0 + a_i + z_i + \epsilon_i$ for different values of the parameter set (σ_a^2, σ_z^2, σ_e^2), with $\beta_0 = 0$ and pedigree and missing structure as in the Swiss barns owl population. The simulated data are fitted to a AZI model (autosomal and Z-linked inheritance) and a AI model (autosomal inheritance). Bias and coverage is reported.

		Fitted model			
		Autosomal and Z-linked inheritance (AZI)		Autosomal inheritance (AI)	
Parameter sets	True values	Bias	Coverage	Bias	Coverage
	$\sigma_a^2 = 0.6$	-0.0010	930	0.0006	931
	$\sigma_z^2 = 0$	0.0045	0	-	-
	$\sigma_e^2 = 0.4$	0.0014	934	0.0016	933
	$\sigma_a^2 = 0.525$	0.0384	781	0.0724	684
	$\sigma_z^2 = 0.1$	-0.0481	403	-	-
	$\sigma_e^2 = 0.4$	0.0021	919	0.0026	924
	$\sigma_a^2 = 0.450$	0.0212	854	0.1430	176
	$\sigma_z^2 = 0.2$	-0.0275	785	-	-
	$\sigma_e^2 = 0.4$	0.0036	930	0.0049	938
	$\sigma_a^2 = 0.375$	0.0062	914	0.2130	13
	$\sigma_z^2 = 0.3$	-0.0080	902	-	-
	$\sigma_e^2 = 0.2$	0.0054	933	0.0081	938
	$\sigma_a^2 = 0.300$	-0.0036	894	0.2862	0
	$\sigma_z^2 = 0.4$	0.0034	929	-	-
	$\sigma_e^2 = 0.4$	0.0042	935	0.0064	939
	$\sigma_a^2 = 0.225$	-0.0126	834	0.3600	0
	$\sigma_z^2 = 0.5$	0.0137	904	-	-
	$\sigma_e^2 = 0.4$	0.0068	918	0.0083	934
	$\sigma_a^2 = 0.150$	-0.0262	753	0.4317	0
	$\sigma_z^2 = 0.6$	0.0275	861	-	-
	$\sigma_e^2 = 0.4$	0.0102	855	0.0092	935
	$\sigma_a^2 = 0.075$	-0.0360	397	0.5039	0
	$\sigma_z^2 = 0.7$	0.0367	821	-	-
	$\sigma_e^2 = 0.4$	0.0160	837	0.0113	926
	$\sigma_a^2 = 0$	0.0038	0	0.5736	0
	$\sigma_z^2 = 0.8$	-0.0027	954	-	-
	$\sigma_e^2 = 0.4$	-0.0003	954	0.0126	921
